# Supplementary material for: c-Met+ Cytotoxic T Lymphocytes Exhibit Enhanced Cytotoxicity in Mice and Humans In Vitro Tumor Models
Source: Biomedicines. 2023 Nov 23;11(12):3123. doi: 10.3390/biomedicines11123123 (PMC10740932; doi:10.3390/biomedicines11123123)
Supplement: Supplementary file 1 [file biomedicines-11-03123-s001.zip › biomedicines-2665578-supplementary.pdf]

Table S1: Antibodies for flow cytometry

Antibodies	Species reactivity	Clone	Fluorochrome	Supplier	RRID number
CD3	Anti-mouse	145-2C11	BV421	BD Horizon	AB_11153670
CD8	Anti-mouse	53-6.7	BUV395	BD Horizon	AB_2732919
c-Met	Anti-mouse	eBioclone7	FITC	Invitrogen	AB_494112
Vβ13	Anti-mouse	MR12-3	APC	Invitrogen	AB_2573224
IFNγ	Anti-mouse	XMG1.2	BUV737	BD Horizon	AB_2870098
Granzyme B	Anti-mouse	NGZB	APC ef780	Invitrogen	AB_2716966
TNFα	Anti-mouse	MP6-XT22	PEcy7	Invitrogen	AB_11042728
CD107a	Anti-mouse	1D4B	BV786	BD Horizon	AB_2738762
KLRG-1	Anti-mouse	2F1	BV421	BD Horizon	AB_2737875
PD-1	Anti-mouse	J43	APC ef780	Invitrogen	AB_2574002
CTLA-4	Anti-mouse	UC10-4F10-11	APC-R700	BD Horizon	AB_2739350
CD178/FasL	Anti-mouse	MFL3	PE	BD OptiBuild	AB_395711
LAG-3	Anti-mouse	eBioC9B7W (C9B7W)	PE	Invitrogen	AB_494214
Tim-3	Anti-mouse	5D12/TIM-3	BV480	BD OptiBuild	AB_2744184
CD3	Anti-human	UCHT1	PE-Cy7	Biolegend	AB_439781
CD8	Anti-human	SK1	APC-Cy7	BD Horizon	AB_2044005
c-Met	Anti-human	95106	FITC	R&D System	AB_1026292
IFNγ	Anti-human	4S.B3	APC	Invitrogen	AB_469506
Granzyme B	Anti-human	GB11	PE	Invitrogen	AB_1659718

Figure S1

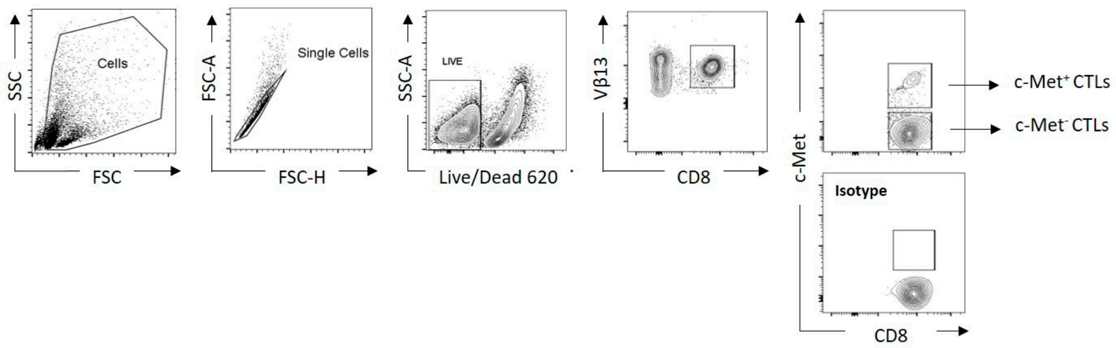

Figure S1. Flow cytometry gating strategy for the identification of Pmel-1 CD8^{+}Vβ13^{+} T cells

Live lymphocytes were first selected for their morphology using FSC/SSC parameters, followed by the exclusion of doublets and dead cells (LD620), and CD8^{+}Vβ13^{+} T lymphocytes were subsequently selected. c-Met expression was defined with FMO control condition.

Figure S2

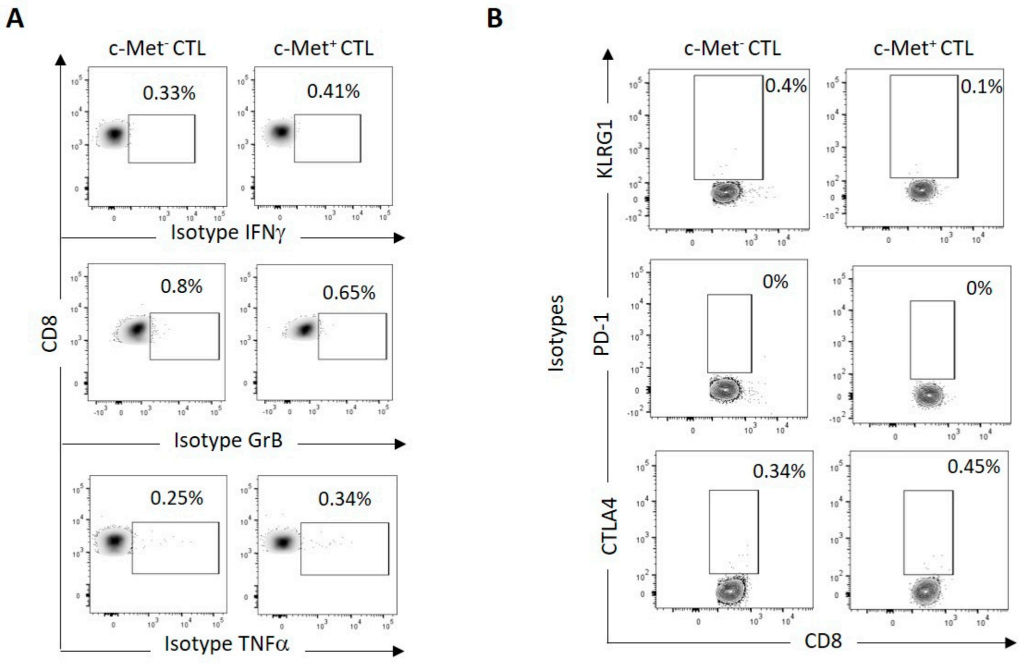

Figure S2. Isotypes controls presentation

Representative density (**A**) and contour plot (**B**) of isotype controls of IFNγ, GrB and TNFα (**A**) and KLRG1, PD-1 and CTLA-4 (**B**).

Figure S3

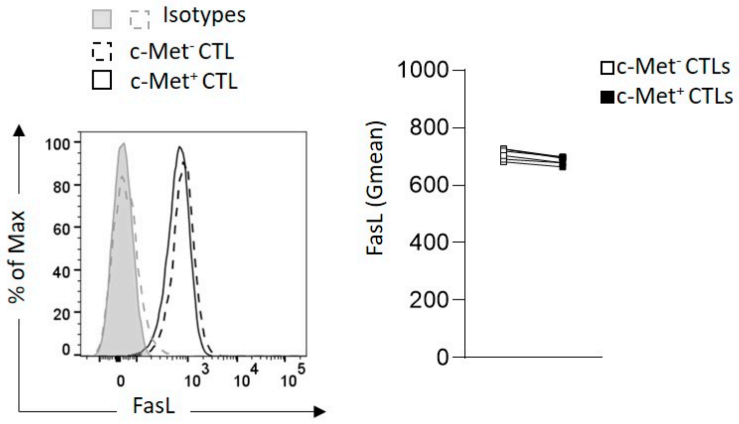

Figure S3. FasL expression by CTLs in contact with B16 Spheroids

Representative histogram (left panel) and paired flow cytometry quantifications (right panel) of FasL 4h after co-culture of B16 spheroids and CTLs c-Met^{+} vs c-Met^{-} from six experiments of (n=6 mice). P-values were calculated using a paired *t*-test.

Figure S4

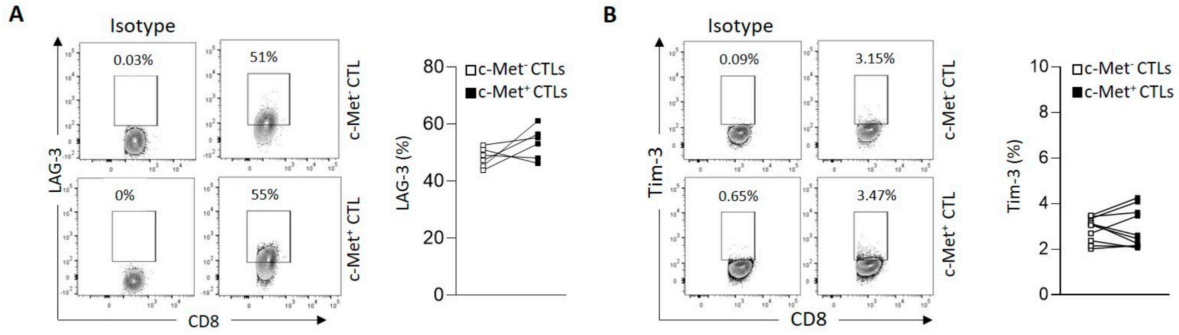

Figure S4. LAG-3 and Tim-3 expression by CTLs in contact with B16 Spheroids

(A, B) Representative contour plots (left panel) and paired flow cytometry quantifications (right panel) of LAG-3 and Tim-3 respectively 4h after co-culture of spheroids B16 and CTLs c-Met^{+} vs c-Met^{-} from six experiments of (n=6 mice). P-values were calculated using paired *t*-test.
